# Society Meetings

**Published:** 1896-04

**Authors:** 


### Society Meetings.

THE International Periodic Congress of Gynecology and Obstetrics will hold its second session at Geneva, Switzerland, during the first week in September, 1896. The official program announces the following subjects for discussion:

In gynecology, (1) Treatment of pelvic suppurations. (2) Surgical treatment of uterine retrodeviations. (3) Which method of suturing the abdomen affords the best guarantee against abscess and hernia?

In obstetrics, (1) Relative frequency and most common forms of pelvic contraction in different races, groups of countries or continents. (2) Treatment of eclampsia.

The subscription to the congress is 30 francs, or $6.00. The secretary for obstetrics is Dr. A. Cordes, of Geneva, and for gynecology, Dr. Betrix, also of Geneva. The American secretary is Dr. F. Henrotin, of Chicago.

---

THE American Medical Publishers' Association will hold its third annual meeting at Atlanta, Ga., Monday, May 4, 1896. A number of new and important topics have been suggested for discussion, and the program will include papers from experienced publishers. Members and others desiring to contribute papers will be furnished valuable information upon communicating with the secretary, Charles Wood Fassett, St. Joseph, Mo. A special invitation has been extended to the association to visit "Lookout Inn," Chattanooga, en route to Atlanta, where entertainment will be provided by the manager, Mr. M. S. Gibson. The association is requested to rendezvous at Chattanooga, Saturday, May 2, 1896.

---

THE National Confederation of State Medical Examining and Licensing Boards will hold its sixth annual meeting at the Hotel

Aragon, Atlanta, Monday, May 4, 1896, beginning at 10 o'clock A. M., under the presidency of Dr. William Warren Potter of Buffalo. An interesting program has been prepared (see page 735), in which among other things it is announced that James Russell Parsons, Jr., Esq., director of examinations of the University of the State of New York, will deliver an address. All physicians and educators friendly to the cause of higher medical education are cordially invited to attend this meeting.

---

The Tri-State Medical Society will hold its annual meeting at Chicago, April 7, 8 and 9, 1896. The program announces, among other things, that Dr. John B. Murphy, of Chicago, will hold a surgical clinic and that Dr. Joseph M. Mathews, of Louisville, will deliver the public address, subject—Some suggestions in regard to reforms in the medical profession. Papers will be read, among others, by the following-named : Dr. A. H. Cordier, Kansas City; Dr. L. H. Dunning, Indianapolis, Dr. Roswell Park, of Buffalo; Dr. D. S. Reynolds, of Louisville, and Dr. Joseph B. Bacon, of Chicago.

---

The American Public Health Association, composed of distinguished hygienists and sanitarians, will hold its next annual meeting in Buffalo during September, 1896. The president is Dr. Eduardo Liceaga, of Mexico, who has written to Dr. Ernest Wende, the local member of the executive committee, that he expects to bring a numerous delegation of his countrymen to attend the congress. The citizens of Buffalo should unite in an endeavor to make this a most superior meeting of the association.

---

The American Academy of Medicine will hold its twenty-first annual meeting at the Hotel Aragon, Atlanta, on Saturday, May 2, and Monday, May 4, 1896, under the presidency of Dr. Henry M. Hurd, of Baltimore. That portion of the program relating to methods of medical teaching is published in another place, page 736, in this issue. The secretary, Dr. Charles McIntire, Easton, Pa., has prepared an elaborate program, and an interesting meeting is expected.

---

The Third International Congress of Dermatology will be held in London, August 4–8, 1896. It is considered of the greatest importance that those intending to join the congress should notify the secretary, Dr. J. J. Pringle, 23 Lower Seymour street, London, W., of their intention soon as possible. The membership fee is $5. Dr. George Thomas Jackson, of New York, is the secretary for the United States.

---

THE Iowa State Medical Society will hold its forty-fifth annual meeting at Des Moines, April 15, 16 and 17, 1896, under the presidency of Dr. Davis S. Fairchild, of Clinton. The secretary, Dr. James W. Cokenower, of Des Moines, has issued an elaborate program that indicates a high grade of professional activity in Iowa.

